# A case of Podocytic Infolding Glomerulopathy with SLE and literature review

**DOI:** 10.1186/s12882-021-02615-4

**Published:** 2021-12-11

**Authors:** Xi Liu, Jieli Huang, Kun Zhang, Yangyang Niu, Yuting Liu, Chunli Cui, Chen Yu

**Affiliations:** The Department of Nephrology, Tongji Hospital, Tongji University School of Medicine, Shanghai, China

**Keywords:** Podocytic infolding glomerulopathy, Podocytes,Systemic lupus erythematosus, EB virus, Ovarian tumor

## Abstract

**Background:**

Podocytic infolding glomerulopathy (PIG) is a rare pathological change which was characterized by the microspheres or microtubular structures in the thickened glomerular basement membrane (GBM). Only a few dozen cases have been reported worldwide so far. Here we present a case of PIG with systemic lupus erythematosus.

**Case presentation:**

A 61-year-old Chinese female was diagnosed with systemic lupus erythematosus with clinical manifestations of proteinuria, pleural effusion, seroperitoneum, anemia, leukopenia, thrombocytopenia, antinuclear antibody positive, and hypocomplementemia. She also had benign ovarian tumor and Epstein-Barr virus infection. Renal biopsy immunofluorescent staining showed IgM and C3 were granularly deposited along the capillary wall instead of typical “full house” features. Electron microscopy showed lots of microspheres structures were seen in the thickened GBM.

**Conclusion:**

We present a case of PIG in a patient with systemic lupus erythematosus. The mechanisms of PIG are unknown, but may be associated with connective tissue disease and podocyte injury.

## Background

Podocytic infolding glomerulopathy (PIG) is a rare pathological changes which was characterized by specific changes to the thickened glomerular basement membrane (GBM), including microspheres, microtubular structures, and podocytic infolding. Only a few dozen cases have been reported worldwide so far. The pathogenesis and clinical characteristics of PIG are still unclear. It is important to accumulate information from reported cases. We present here a case of PIG with systemic lupus erythematosus.

## Case presentation

The patient was a 61-year-old female, from Sichuan Province, China. She had a history of hypertension for five years and underwent a cholecystectomy for gallstones ten years ago. The patient was initially presented to the department of gastroenterology because of abdominal distention and poor appetite. She had dry mouth and several teeth loss. In the past six months she had lost 10 kg weight. She underwent colonoscopy and capsule endoscopy and no abnormalities were observed in the intestinal mucosa. B-ultrasound examination suggested a big pelvic cystic mass, about 75*65*64 mm in size, accompanied by pelvic effusion (depth of about 86 mm), abdominal effusion (right upper abdomen 97 mm, left upper abdomen 46 mm), and bilateral pleural effusion (depth of about 36 mm). Then the patient was admitted to the gynecological ward and received bilateral adnexectomy and peritoneal biopsy under laparoscopy. Pathological examination confirmed benign serous cystadenoma of ovary. Meanwhile, the patient’s blood and urine tests showed abnormalities. She was diagnosed with anemia, leukopenia, thrombocytopenia hypoproteinemia and proteinuria. Immunological examination showed that the patient was positive for multiple autoantibodies including ANA, anti-dsDNA, anti-SSA, anti-RNP, anti-mitochondrial M2 and anti-ribonucleoprotein. Coombs test (both direct and indirect method) was negative. Reticulocyte was normal (1.24%).Bone marrow puncture suggested bone marrow hyperplasia was active. Serum EB virus DNA quantification was 2.8*10^3^copies/mL.Laboratory data was showed in Table 1.

**Table 1:** Laboratory data before the renal biopsy

Item	Value
Creatinine	0.85 mg/dl
Uric Acid	580 umol/l
Hb	82 g/L
RBC	2.81*10^12^/L
WBC	2.89*10^9^/L
PLT	110*10^9^/L
Albumin	26.5 g/L
Cholesterol	3.94 mmol/L
Triglyceride	3.41 mmol/L
24 h UTP	2.06 g/day
URBC	19/ul
UWBC	132/ul
ANA	1:320
Anti-dsDNA	129.071 IU/ml
Anti-SSA	positive
Anti-RNP	positive
Anti-mitochondrial M2	positive
Anti-ribonucleoprotein	positive
C3	0.25 g/L
C4	0.06 g/L
Anti-GBM	negative

*Hb* Hemoglobin, *RBC* red blood cells, *WBC* white blood cells, *PLT* platelets

*UTP* urinary total protein, *URBC* urinary red blood cells

*UWBC* urinary white blood cells, *ANA* antinuclear antibody

Then she was diagnosed with systemic lupus erythematosus. The renal biopsy was performed. Renal biopsy findings are shown below.

**Fig. 1:**
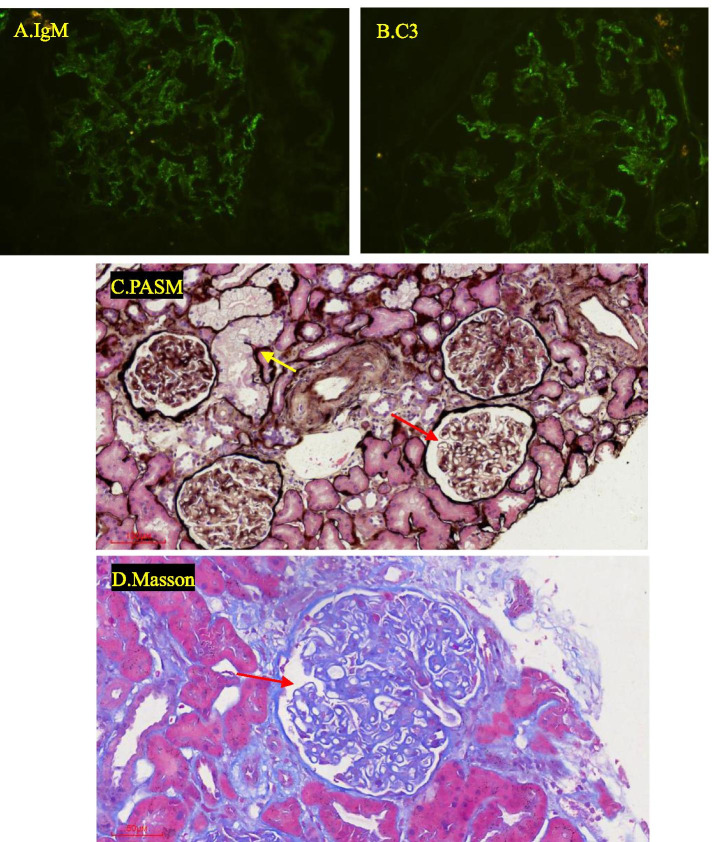
Renal biopsy findings (A ~ D) A. IgM (400X); B. C3(400X); C.D. Thickened capillary (→) and tubular epithelial cells vacuolar degeneration (→)

Immunofluorescent staining: IgG -, IgA -, IgM ++, C3 ++, C1q -, Kappa +, Lambda ++, granularly deposition along the capillary wall (Fig. 1 A,B).

Light microscopy: A total of 20 glomeruli and 5 glomeruli were global sclerosis. Mesangial cells and matrix mildly increased, capillary walls was thickened (Fig. 1C,D). Vacuolar degeneration was observed in renal tubular epithelial cells (Fig. 1C). A few tubules were atrophic along with slight interstitial fibrosis. There were a few inflammatory cells infiltrating in the renal interstitial. Arteriole intima was thickened without hyaline degeneration.

**Fig. 2:**
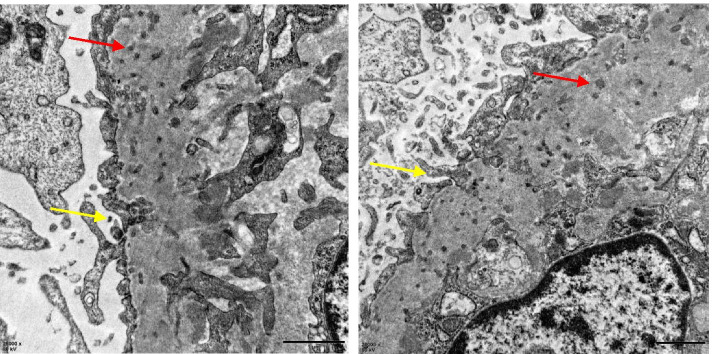
Microspheres in the thickened GBM (→) and podocytes components trapped in the GBM (→)

Electron microscopy: The foot processes were extensively fused and GBM diffusely thickened. A large number of microspheres were found in GBM and podocytes infolded into the basement membrane (Fig. 2). Reticular aggregate was seen in the cytoplasm of endothelial cells.

Finally the patient was diagnosed with lupus nephritis and PIG. We gave her losartan (100 mg daily), hydroxychloroquine (100 mg twice a day), methylprednisolone (40 mg daily) and cyclophosphamide (600 mg monthly) treatment. Two weeks later, serum albumin was increased to 31.7 g/ L, serum creatinine was 83umol/ L, hemoglobin was 77 g/L, red blood cell 2.55*10^12^/L, white blood cell 3.02*10^9^/L and platelet 155*10^9^/L.Then the patient was discharged to her hometown for further treatment.

## Discussion and conclusions

PIG is a rare pathological change and mainly reported in East Asian countries. In recent years, it has also been reported in European countries [1]. It was characterized by the microspheres or microtubular structures in the GBM and usually coexisted with autoimmune diseases. It was also reported in membranous nephropathy, hepatitis B, malignant tumors [2, 3]. In a few cases, PIG appears alone. But whether it is an independent disease is controversial.

The pathogenesis and clinical characteristics of PIG are unknown. The current understanding is limited to ultrastructural morphological observations. Podocytes can produce type IV collagen, laminin, and heparin sulfate to participate in the formation of GBM, but the arrangement and degradation of the GBM is still unclear [4]. Endothelial cells are also involved in GBM synthesis, but it is not clear whether endothelial cells are related to PIG. Some researches found that intra-GBM microstructures was vimentin (a marker for both podocyte and endothelium) and C5b-9 [5]. Genetic mutations may also be involved in PIG. Recent case report showed PIG with novel SMARCAL1 mutations [6]. There is no consensus on the treatment of PIG. Glucocorticoid therapy can decreased proteinuria, suggesting that the mechanism of PIG may be related to immune disorders and podocyte injury. The effect of PIG on the prognosis of patients is not clear.

There are several differences in our case: Firstly, the patient had dry mouth, several teeth loss, anti SSA antibody positive and mitochondria antibody positive, but the patient finally rejected the labial gland biopsy and liver puncture, so we can’t rule out the sjogren’s syndrome and autoimmune hepatitis. Second, the patient had benign serous cystadenoma of ovary and the Epstein-Barr virus DNA copy number was elevated, suggesting that tumor or viral infection may be involved in PIG. Third, the renal pathology of the patient was not the typical “full house”, and lacked the deposition of electron dense material in the subcutaneous, subepithelial and mesangial areas, which was different from the pathological changes of classical lupus nephritis.

## Data Availability

The data used to support the findings of this study are available from the corresponding author upon request.
